# Clinical factors associated with circulating tumor DNA (ctDNA) in primary breast cancer

**DOI:** 10.1002/1878-0261.12456

**Published:** 2019-02-06

**Authors:** Yidong Zhou, Yaping Xu, Yuhua Gong, Yanyan Zhang, Yaping Lu, Changjun Wang, Ru Yao, Peng Li, Yanfang Guan, Jiayin Wang, Xuefeng Xia, Ling Yang, Xin Yi, Qiang Sun

**Affiliations:** ^1^ Department of Breast Surgery Peking Union Medical College Hospital Chinese Academy of Medical Sciences Beijing China; ^2^ Geneplus‐Beijing Institute China; ^3^ Department of Computer Science and Technology School of Electronic and Information Engineering Xi'an Jiaotong University China

**Keywords:** circulating cell‐free DNA, clinical factors, concordance, next‐generation sequencing, primary breast cancer

## Abstract

Noninvasive circulating tumor DNA (ctDNA) can be used to predict breast cancer recurrence and prognosis. In this study, we detected 226 and 114 somatic variants in tumor DNA from 70 primary breast cancer (PBC) patients (98.59%) and ctDNA from 48 patients (67.61%), respectively. Gene frequencies of tumor DNA and ctDNA significantly correlated (*R* ^2 ^= 0.9532, *P *< 0.0001), and tumor‐derived variants were detectable in the blood of 43 patients. ctDNA was more often detected in locally advanced/metastatic and nonluminal patients. Multivariate analysis revealed that individual N stage (*P *<* *0.001) and hormone receptor (HR) status (*P *=* *0.001) could independently predict the detectability of tumor‐derived mutations in blood. The maximal variant allele frequency of ctDNA was significantly higher in patients with stage IV/M1 (*P *=* *0.0136) and stage T3/T4 (*P *=* *0.0085) cancers. Finally, clonal variants in tumor DNA were more easily traced in ctDNA than subclonal variants (84.62% vs 48.75%). In conclusion, ctDNA fragments concordant with tumor DNA can be consistently detected in the majority of tested PBC patients, which may enable noninvasive genomic profiling of PBC, particularly for patients with advanced‐stage tumors and positive HR status.

**Abbreviations**
CA15‐3: cancer antigen 15‐3
CCF: cancer cell fraction
CEA: carcinoembryonic antigen
cfDNA: cell‐free DNA
CNV: copy‐number variant
COSMIC: Catalogue of Somatic Mutations in Cancer
ctDNA: circulating tumor DNA
ER‐Seq: enrich rare mutation sequencing
GO: Gene Ontology
HR: hormone receptor
KEGG: Kyoto Encyclopedia of Genes and Genomes
MVA: multivariate analysis
NGS: next‐generation sequencing
PBC: primary breast cancer
PBL: peripheral blood lymphocyte
SNV: single‐nucleotide variant
TCGA: The Cancer Genome Atlas
UID: unique identifier
UVA: univariate analysis
VAF: variant allele frequency

## 1 Introduction

Breast cancer is the most commonly diagnosed cancer in women worldwide and is the leading cause of cancer‐related deaths in both developed and developing countries (Torre *et al*., 2015). Recently, clinical treatment of primary breast cancer (PBC) has been advanced by tissue‐based and large‐scale genomic profiling, although the results of these techniques are limited by sampling bias and tumor heterogeneity (Zardavas *et al*., 2015). Moreover, tissue biopsies cannot fully monitor the dynamic changes in tumor burden, and circulating biomarkers currently used, including carcinoembryonic antigen (CEA) and cancer antigen 15‐3 (CA15‐3), have low sensitivity in some patient populations (Wu *et al*., 2014). Thus, novel biomarkers that can be assayed noninvasively provide real‐time monitoring of tumor burden and sensitively reflect tumor molecular characteristics are urgently needed for effective clinical cancer treatment.

One potential biomarker is circulating tumor DNA (ctDNA), which is derived from necrosis, apoptosis, and secretions of tumor cells and can be detected in peripheral blood. Importantly, the detectability of ctDNA diverse solid tumors has been validated (Schwarzenbach *et al*., 2011), including that of PBC (Garcia‐Murillas *et al*., 2015; Higgins *et al*., 2012; Olsson *et al*., 2015; Schiavon *et al*., 2015). However, detection of ctDNA mutations is difficult in some patients, especially those with early‐stage cancer (Bettegowda *et al*., 2014). Furthermore, data regarding the influence of clinical factors on ctDNA detection are limited, and the correlation between mutations in tumor DNA and ctDNA is still unknown.

In this study, we thoroughly characterized the relationship between mutations in tumor DNA and ctDNA and analyzed how ctDNA profiling is affected by various clinical factors. We collected paired tumor tissue and peripheral blood samples from 71 recently diagnosed PBC patients and applied panel‐based next‐generation sequencing (NGS) to evaluate the genomic variants in all specimens. We made novel comparisons of mutation prevalence between tumor DNA and ctDNA and collected detailed clinical information to explore the potential factors affecting the detectability of circulating tumor‐derived genetic variations.

## 2 Materials and methods

### 2.1 Clinical cohort

In this prospective cohort study, 71 patients recently diagnosed with PBC at Peking Union Medical College Hospital from May 2016 to November 2016 were enrolled (ClinicalTrials.gov Identifier: NCT02797652). Clinicopathological data regarding demography and tumor histopathological results, such as TNM staging and immunohistochemical information, were collected from each patient. TNM staging was defined according to the American Joint Committee on Cancer TNM staging system for breast cancer (Singletary *et al*., 2002). Patient HER2 status was initially evaluated by immunohistochemical staining (Ventana 4B5 antibody and BenchMark XT; Roche, Basel, Switzerland). In patients with 2+ status, fluorescence *in situ* hybridization was performed to further determine the expression of HER2 (Vysis probe; Abbott, Chicago, IL, USA). All procedures were executed by at least two experienced pathologists who adhered to the American Society of Clinical Oncology/College of American Pathologists’ clinical practice guidelines (Wolff *et al*., 2013). The molecular subtype of each tumor was defined using immunohistochemical parameters as previously described (Goldhirsch *et al*., 2013). Three healthy individuals were also recruited as control participants to test the accuracy of our sequencing platform for ctDNA profiling. This study was approved by the ethical committee at Peking Union Medical College Hospital. All participants provided informed written consent before undergoing any study‐related procedures. This study was performed in accordance with the Declaration of Helsinki.

### 2.2 Sample collection and DNA extraction

Before treatment, tumor tissue samples of each patient were prospectively obtained via coarse needle aspiration, and immunohistochemical analysis was used to quantify expression of hormone receptor (HR), HER2, and Ki67. Fewer than 4 days after tissue sampling, peripheral blood samples of at least 10 mL were also collected in Streck tubes from all patients and healthy controls. Within 3 days of collection, peripheral blood was separated by centrifugation at 1600 ***g*** for 10 min, transferred to microcentrifuge tubes, and centrifuged again at 16 000 ***g*** for 10 min to remove cell debris. Tissues, plasma, and peripheral blood lymphocytes (PBLs) were stored at −80 °C prior to DNA extraction. Genomic DNA was extracted from tumor tissue (tumor DNA) and PBL (germline DNA) using a QIAamp DNA Mini Kit and QIAamp DNA Blood Mini Kit (Qiagen, Hilden, Germany), respectively. Circulating cell‐free DNA (cfDNA) was extracted from plasma using the QIAamp Circulating Nucleic Acid Kit (Qiagen). DNA concentration was estimated using a Qubit fluorometer and a Qubit dsDNA high sensitivity (HS) Assay Kit (Invitrogen, Carlsbad, CA, USA). cfDNA fragment length was assessed using an Agilent 2100 Bioanalyzer and the DNA HS kit (Agilent Technologies, Santa Clara, CA, USA).

### 2.3 Library preparation

Tumor and germline DNA were sheared into 200‐ to 250‐bp fragments using a Covaris S2 instrument (Woburn, MA, USA), and indexed NGS libraries were prepared using the DNA Library Preparation Kit for Illumina (New England Biolabs, Ipswich, MA, USA). For cfDNA, after end‐repairing and A‐tailing reactions, targeted adapters with unique identifiers (UIDs) were ligated to both ends of double‐stranded cfDNA fragments, followed by PCR to generate sufficient numbers of fragments prior to hybridization. Additional detailed information regarding library preparation was described by Lv *et al*. (2017).

### 2.4 Target region capture and next‐generation sequencing

All libraries were hybridized to custom‐designed biotinylated oligonucleotide probes (IDT, Coralville, IA, USA) covering 1.09 Mbp of the genome. The captured genomic regions included the most common driver genes of solid tumors (Kandoth *et al*., 2013). We chose their entire exome regions to construct the basic panel. Next, genomic regions related relevant to the effects of chemotherapy, targeted drugs, and immunotherapy per available clinical and preclinical research were added to the panel. Finally, high‐frequency mutant regions recorded in the Catalogue of Somatic Mutations in Cancer (COSMIC, http://cancer.sanger.ac.uk/cosmic) and The Cancer Genome Atlas (TCGA, https://cancergenome.nih.gov/) were involved. All included genes are shown in Table S1. DNA sequencing was performed using an Illumina 2 × 75‐bp paired‐end sequencing strategy on the HiSeq Sequencing System (Illumina, San Diego, CA, USA), which generated 1, 2, or 15 Gb of data from germline DNA, tumor DNA, or cfDNA, respectively. Additional detailed information regarding target region capture and NGS was described by Lv *et al*. (2017).

### 2.5 Raw data processing

After removing raw reads containing adaptor sequences, those with more than 50% low‐quality base reads, or those with more than 50% N bases, together with their mate pair, reads were mapped to the reference human genome (hg19) using the Burrows‐Wheeler Aligner (http://bio-bwa.sourceforge.net/) with default parameters. Duplicate reads were identified and marked with Picard's Mark Duplicates tool (https://software.broadinstitute.org/gatk/documentation/tooldocs/4.0.3.0/picard_sam_markduplicates_MarkDuplicates.php) for tumor and germline DNA data and were clustered according to UID and position of the template fragments for cfDNA data. Errors introduced by PCR or sequencing were corrected according to clustered reads. Local realignment and base quality recalibration were performed using The Gene Analysis Toolkit (https://www.broadinstitute.org/gatk/).

### 2.6 Somatic mutation calling of tumor DNA

Somatic single‐nucleotide variations (SNVs) were called using the MuTect2 algorithm (https://software.broadinstitute.org/gatk/documentation/tooldocs/3.8-0/org_broadinstitute_gatk_tools_walkers_cancer_m2_MuTect2.php). Candidate mutations were filtered if (a) more than 10 reads with insertions/deletions in an 11‐bp window were centered; (b) the matched germline DNA control sample carried ≥ 3% or ≥ 2% alternate allele reads, and the sum of quality scores was above 80; (c) the candidate was found in dbsnp (version 138, https://www.ncbi.nlm.nih.gov/SNP/) but not listed in the COSMIC database; (d) the candidate was supported by fewer than five high‐quality reads (base quality ≥ 30, mapping quality ≥ 30); or (e) the allele frequency was < 1%. Insertions or deletions of small fragments (indels) were called using MuTect2 with default parameters. Variants detected in matched control samples with three or more reads indicating indels at the same location or in the 40‐bp flanking regions of experimental samples or residing near regions with low complexity or short tandem repeats were removed. Remaining mutations were considered validated somatic variants.

### 2.7 Enrich rare mutation sequencing (ER‐Seq) of ctDNA

For somatic SNVs/indels of ctDNA, the accuracy of low‐frequency mutations was further confirmed by high‐quality support reads with both forward and reverse strand read pairs clustered by UID and filtration through the control group (germline mutations), as well as screening for ‘background noise’ false‐positive ctDNA mutations. The enrich rare mutation sequencing (ER‐Seq) strategy combines the following features: (a) unique sequencing adapters and bidirectional error correction; (b) a capture panel that covers over 95% of tumor‐related mutations from common cancers; and (c) background noise database filtering (Lv *et al*., 2017). As a result, ER‐Seq compensates for errors introduced by PCR and/or sequencing and enables efficient and precise detection of rare mutations in blood samples. For example, the minimal allelic fraction reliably detectable in ctDNA is 0.5%.

### 2.8 Somatic copy‐number variation (CNV) calling

The CONTRA algorithm (http://contra-cnv.sourceforge.net) was used to detect copy‐number variants (CNVs) in tumor DNA and cfDNA.

### 2.9 Enrichment and clonality analyses

For enrichment analysis of mutated genes, the Gene Ontology (GO; http://www.geneontology.org/) and Kyoto Encyclopedia of Genes and Genomes (KEGG; http://www.genome.jp/kegg/pathway.html) tools were applied for various data subsets. To classify variants identified in tissue as clonal or subclonal mutations, the cancer cell fraction (CCF) was defined using mutant variant allele frequency (VAF) (Roth *et al*., 2014). Mutations with maximal CCF and CCF ≥ 75% of maximum were classified as clonal; all others were classified as subclonal.

### 2.10 Statistical analysis

A chi‐squared test was performed using spss 22.0 (IBM, Armonk, NY, USA) to compare the mutation rates of *PIK3CA* between HR‐positive and HR‐negative patients, as well as the detection rate of ctDNA between patients with different TNM stages and molecular subtypes. Pearson correlation analysis was performed using graphpad prism 6 (GraphPad Software, La Jolla, CA, USA) to assess the relevance of mutated gene frequencies between tumor DNA and ctDNA. To analyze the correlation between different clinical features and the positive detection rate of ctDNA, univariate analysis (UVA) and multivariate analysis (MVA) were performed using spss 22.0. The relationship between maximal VAF (MVAF) and different clinical features was assessed by one‐way ANOVA with spss 22.0. A *P* value < 0.05 indicated statistical significance.

## 3 Results

### 3.1 Patient characteristics and sequencing quality control

A summary of PBC patient characteristics is found in Table 1. Patients were diverse in both diagnostic age and disease parameters. For example, the median diagnostic age of patients was 47 years (range, 27–78 years); 11 (15.49%) patients were younger than 35 years, 42 (59.16%) were between 35 and 55 years, and the remaining 18 (25.35%) were older than 56 years. Over half patients (53.52%) were TNM stage III, and the proportions of patients with stages I, II, and IV were 11.27%, 25.35%, and 9.86%, respectively. Twelve patients (16.90%) had either a maximal tumor diameter > 5 cm (T3 stage) or chest wall and skin invasion (T4 stage), while lymph node metastasis occurred in 52 patients (73.24%, N1–N3 stage). Fifty‐two (73.24%) patients were HR‐positive, and 28 (39.44%) overexpressed HER2. The Ki67 levels of 60 (84.51%) patients were higher than 14%. The proportions of patients with molecular subtypes of Luminal A, Luminal B, HER2 overexpression, and triple‐negative breast cancer (TNBC) were 4.23%, 67.61%, 12.68%, and 14.08%, respectively.

**Table 1:** Clinical characteristics of study patients with PBC

Characteristics	Total (*n* = 71)
Diagnostic age (years)	
Median (range)	49 (27–78)
Stage (%)	
I	8 (11.27)
II	18 (25.35)
III	38 (53.52)
IV	7 (9.86)
T stage (%)	
T1	26 (36.62)
T2	33 (46.48)
T3	9 (12.68)
T4	3 (4.23)
N stage (%)	
N0	19 (26.76)
N1	12 (16.90)
N2	20 (28.17)
N3	20 (28.17)
HR status (%)	
Positive	52 (73.24)
Negative	19 (26.76)
HER2 overexpression (%)	
Positive	28 (39.44)
Negative	42 (59.15)
Unknown	1 (1.41)
Ki67 levels (%)	
High (> 14%)	60 (84.51)
Low (≤ 14%)	11 (15.49)
Molecular subtypes (%)	
Luminal A	3 (4.23)
Luminal B	48 (67.61)
HER2 overexpression	9 (12.68)
TNBC	10 (14.08)
Unknown	1 (1.41)

Cell‐free DNA was successfully extracted from all patients and healthy individuals. The average concentration of cfDNA was 7.23 ng·mL^−1^ (range, 2.74–20.09 ng·mL^−1^, Table S2). The length of cfDNA fragments primarily ranged from 170 to 185 bp, although another minor peak was centered at ~ 322 bp (Fig. S1). The average coverage depths without duplicate reads for sequenced tumor DNA and cfDNA were 785.63× (range, 425.25× to 1519.92×) and 1006.88× (range, 280.61× to 1669.07×), respectively, and the fractions of target region coverage were all above 99% (Fig. S2, Table S2). For control blood samples, the average sequencing depth was 1012.82× (range, 960.07× to 1042.27×), and their coverage fractions were also above 99% (Fig. S2, Table S2). The sequencing parameters demonstrated no discrepancy between control and experimental blood samples.

### 3.2 Mutation prevalence in tumor DNA and ctDNA

**Figure 1:**
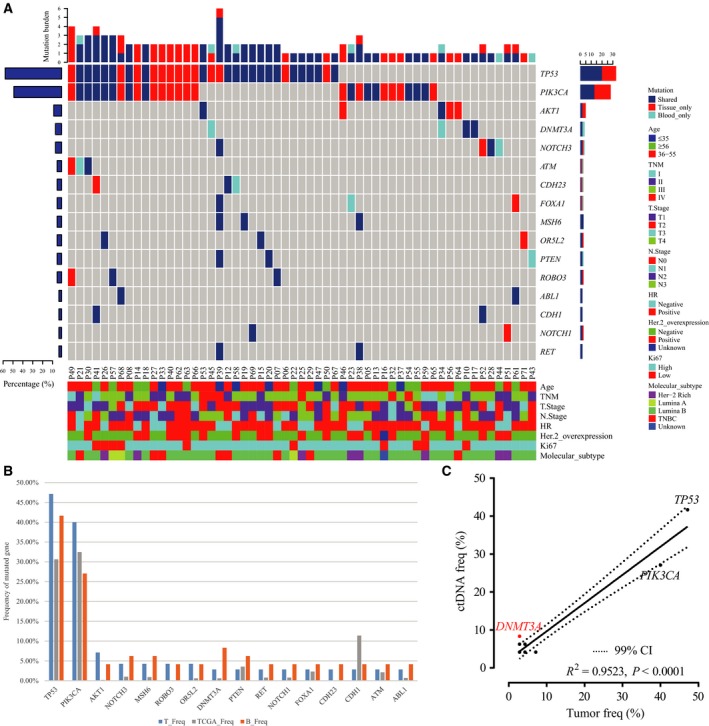
Genomic profiling of tumor DNA, ctDNA, and public database sequences reveals strong associations between mutations in tumor DNA and ctDNA. (A) Prevalence of mutated genes in tumor DNA and ctDNA. The top 16 genes mutated in ctDNA are listed. The bar chart below shows the clinical features of patients, while the bar chart above shows the number of genes altered in each patient. The right bar represents the frequency of specific altered genes in the total cohort. (B) Comparison of gene mutation rates in tumor DNA, ctDNA, and TCGA sequences (SNVs and insertions/deletions). (C) Correlation between mutation rates in ctDNA and tumor DNA.

We detected 226 SNVs, as well as indels in 70 tumors (98.59%) with a median of 2.5 per sample (range, 1–15). We observed no mutations in control blood, indicating the satisfactory specificity of our ctDNA sequencing platform. In comparison, sequencing of experimental blood samples revealed that 48 (67.61%) patients collectively had 114 SNVs and indels with a median of two per sample (range, 1–7). In tumor DNA, genes with the most recurrent somatic mutations were *TP53* (47.14%), *PIK3CA* (40.00%), and *AKT1* (7.14%), while the most frequently mutated genes of ctDNA were *TP53* (41.67%), *PIK3CA* (27.08%), and *DNMT3A* (8.33%; Fig. 1A, Fig. S3). Notably, the mutation rate of *PIK3CA* in tumor DNA and/or ctDNA of HR‐positive patients was 48.08%, while a much lower rate was observed for HR‐negative patients (15.79%; *P *= 0.014; Fig. 1A).

To confirm the validity of our sequencing results, we next compared the prevalence of SNVs and indels in our cohort to those detected in TCGA (*n* = 1105), a publicly available tissue‐based database. For consistency, the analysis of both cohorts was adjusted to only include genes mutated in the ctDNA of more than two patients. Thus, we included 16 genes from our cohort, most of which were present in TCGA. Interestingly, the gene mutation rates reported for the ctDNA from our cohort were generally higher than those in TCGA, except for *PIK3CA* and *CDH1*, possibly due to the low depth of whole exon sequencing data in TCGA (Fig. 1B).

Because tissue‐based sequencing remains the most reliable method for analyzing the molecular features of solid tumors, we also assessed the correlation between the prevalence of mutations in ctDNA and tumor DNA in our cohort and found their mutation rates to be strikingly similar (Fig. 1B). In addition, the mutation rates of the 16 most frequently mutated genes in ctDNA significantly correlated with those in tumor DNA (*R* ^2 ^= 0.9235; *P *< 0.0001; Fig. 1C). Thus, detection of mutations in ctDNA can accurately reflect mutation rates in tumor DNA. We also observed differences between ctDNA and tumor DNA. For example, the detection rate of ctDNA *DNMT3A* mutations was dramatically higher than in tumor DNA (8.33% vs 2.86%; Fig. 1C), a phenomenon that could be attributed to clonal hematopoiesis, which intrinsically leads to substantial noncancerous mutations in blood circulation (Xie *et al*., 2014).

**Figure 2:**
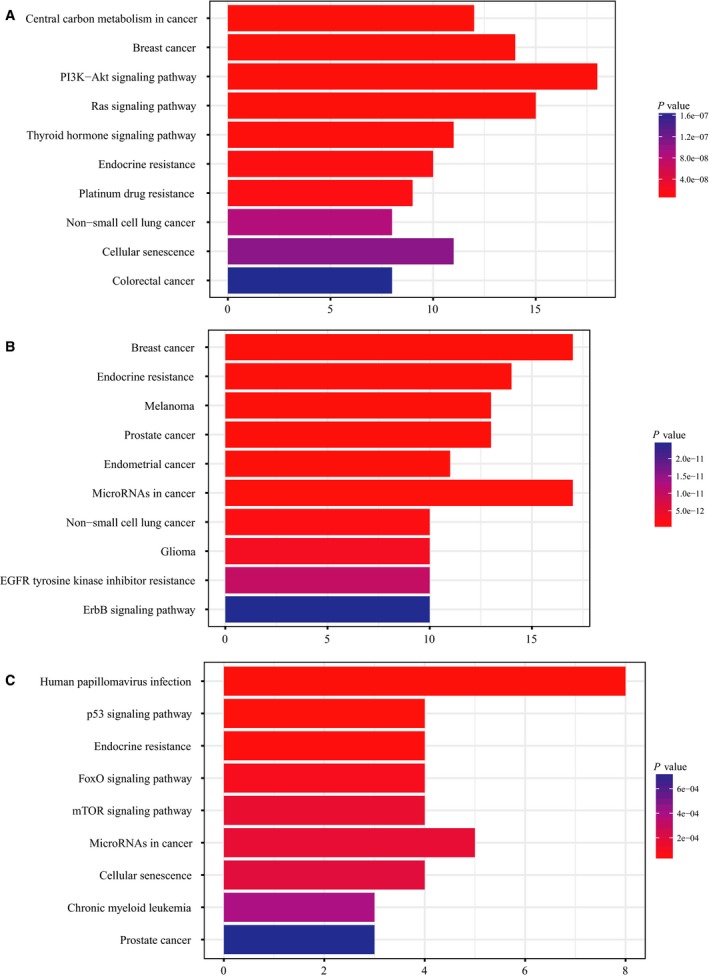
KEGG analysis of biological relevance for tissue‐specific, blood‐specific, and overlapping mutations. (A) KEGG analysis for tissue‐specific mutated genes. (B) KEGG analysis for overlapping mutated genes. (C) KEGG analysis for blood‐specific mutated genes. The length of each column represents the number of enriched genes, and shading of bars indicates statistical significance.

>Except for one patient without any tissue DNA mutations, tumor‐derived mutations could be fully traced in cfDNA of 15 patients (21.43%) and partially traced (26.67–80.00%) in ctDNA of 28 patients (40.00%). For 48 patients with somatic mutations in ctDNA, we detected 154 mutations in tumor tissue, among which 94 mutations (61.03%) could be detected in matched blood, whereas 60 mutations (38.97%) were exclusively detected in tumor DNA. Additionally, 20 mutations were exclusively detected in ctDNA (Fig. S3). To explore the biological features associated with the different mutation subsets (e.g., tissue‐specific, blood‐specific, and overlapping mutations), we performed GO and KEGG enrichment analyses. We selected the top 10 objects for further study, which revealed that tissue‐specific and overlapping mutations possibly co‐altered 17 genetic functions, including phosphatidylinositol‐mediated signaling, receptor complex formation, and tyrosine kinase activity, while blood‐specific mutations exhibited fairly distinct effects compared with the other subsets (Fig. S4). According to KEGG analysis, ‘breast cancer’ pathway is significantly affected by both tissue‐specific and overlapping mutations (Fig. 2A,B) but not blood‐specific mutations. Several enriched pathways related with drug resistance and sensitivity, including endocrine resistance, epidermal growth factor receptor tyrosine kinase inhibitor resistance, and ErbB signaling pathways, were highlighted with respect to overlapping mutations (Fig. 2B). However, the affected pathways enriched in blood‐specific mutations were atypical for breast cancer, although some pathways were indeed meaningful to breast cancer, such as p53 signaling pathway, endocrine resistance, and mTOR signaling pathway (Fig. 2C). The pathway related with ‘human papillomavirus infection’ may originate from the randomness of mutant genes. The fact that such enriched pathway is absent in tissue‐specific and overlapping mutations may confirm our conjecture to some extent. Together, these data indicate that tracking tumor‐derived mutations in ctDNA may enable effective profiling of tumor molecular features and management of targeted agents for PBC patients. However, the genetic implications of blood‐specific mutations relevant to PBC remain unclear.

### 3.3 Clinicopathological parameters associated with the detectability of tumor‐derived mutations in blood

**Figure 3:**
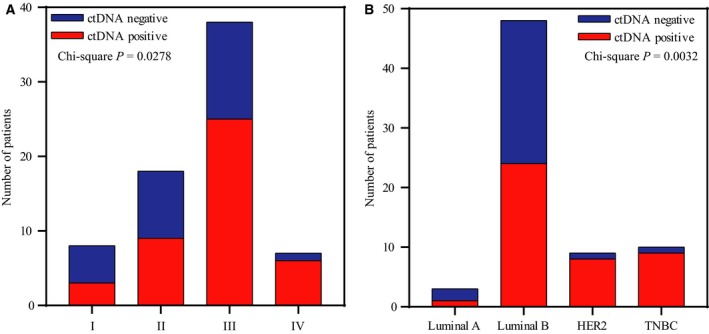
Effect of TNM stage and molecular subtype on detectability of ctDNA. Chi‐squared analysis of the detection rate of ctDNA in patients with (A) different TNM stages and (B) different tumor molecular subtypes.

Based on the above results, blood‐specific mutations were excluded in subsequent analysis, and the association between clinicopathological features and detectability of tumor‐derived variations in blood was evaluated. First, we performed chi‐square tests to determine the effects of TNM staging or molecular subtype on ctDNA detection, which showed statistically significant discrepancies between patients with different stages (chi‐square *P *= 0.0278) and subtypes (chi‐square *P *= 0.0032). In addition, ctDNA was more often detected in locally advanced/metastatic and nonluminal patients than others (Fig. 3). To further clarify the effects of these clinical parameters, individual T stage (T), nodal (N stage), metastasis (M stage), HR, HER2, and Ki67 status, all of which were intrinsic factors in TNM staging and molecular subtyping, as well as diagnostic age of patients, were included in UVA and MVA of ctDNA detection. UVA revealed that tumor‐derived variations were more prevalent in blood of patients with lymph node metastasis (N1–N3) than in lymph node‐negative (N0) patients (69.23% vs 36.84%, *P *< 0.001; Table 2). HR‐positive patients presented with fewer tumor‐derived variations in blood than HR‐negative patients (50.00% vs 89.47%; *P *= 0.002; Table 2). Moreover, MVA showed that individual N stage (*P *< 0.001) and HR status (*P *= 0.002) could independently predict the detection success of tumor‐derived variations in blood (Table 2). Notably, the positive rate of ctDNA in M1 patients exceeded that of M0 patients (85.71% vs 57.81%, Table 2), although the discrepancy did not reach statistical significance, possibly due to the small number of M1 patients in our study.

**Table 2:** Univariate and multivariate analyses of clinical characteristics influencing ctDNA detection

Characteristics	Group	*n*	ctDNA‐positive^a^ *n* (%)	*P* value	
				UVA	MVA
Diagnostic age (years)	≤ 35	12	5 (41.67)	0.520	0.511
	36–55	40	27 (67.50)		
	≥ 56	19	11 (57.89)		
T stage	T1	26	12 (46.15)	0.087	0.068
	T2	33	22 (66.67)		
	T3	9	7 (77.78)		
	T4	3	2 (66.67)		
N stage	N0	19	7 (36.84)	< 0.001^b^	< 0.001^b^
	N1	12	5 (41.67)		
	N2	20	13 (65.00)		
	N3	20	18 (90.00)		
M stage	M0	64	37 (57.81)	0.156	0.147
	M1	7	6 (85.71)		
HR status	Positive	52	26 (50.00)	0.002^b^	0.002^b^
	Negative	19	17 (89.47)		
HER2 overexpression	Positive	28	18 (64.29)	0.504	0.557
	Negative	41	23 (56.10)		
Ki67	High	60	37 (61.67)	0.662	0.493
	Low	11	6 (54.55)		

a ctDNA was defined as positive if tumor‐derived mutations could be detected in blood.

b Represents statistical significance.

**Figure 4:**
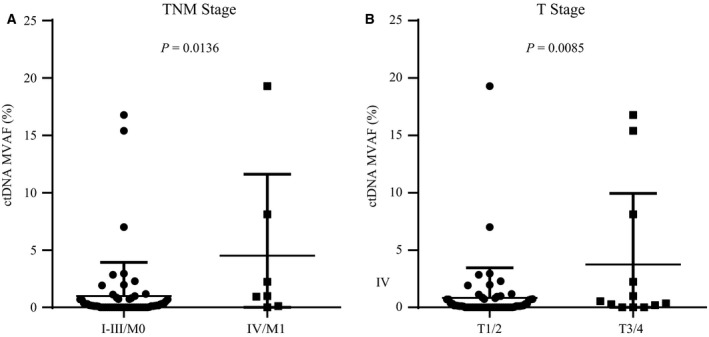
Comparative analysis of ctDNA MVAF between different cancer subgroups with respect to T and TNM staging. (A) The comparative analysis of ctDNA MVAF between (A) early‐ and late‐stage cancer and (B) in patient groups with different T stages.

The VAF of ctDNA may represent molecular tumor burden and thus be utilized in tumor surveillance (Zhou *et al*., 2016). There, we also evaluated the influence of different clinical factors on the MVAF of ctDNA and found that MVAF was significantly higher in metastatic patients (stages IV/M1) than in early‐stage or locally advanced patients (stage I to III/M0; *P *= 0.0136, Fig. 4A). MVAF also positively correlated with advanced T stage (*P *= 0.0085, Fig. 4B), but we saw no statistically significant correlation between MVAF and N stage, diagnostic age, HR or HER2 status, Ki67 level, or molecular subtype (Fig. S5).

### 3.4 Effect of tumor clonality on detection of tumor‐derived variations in blood

**Figure 5:**
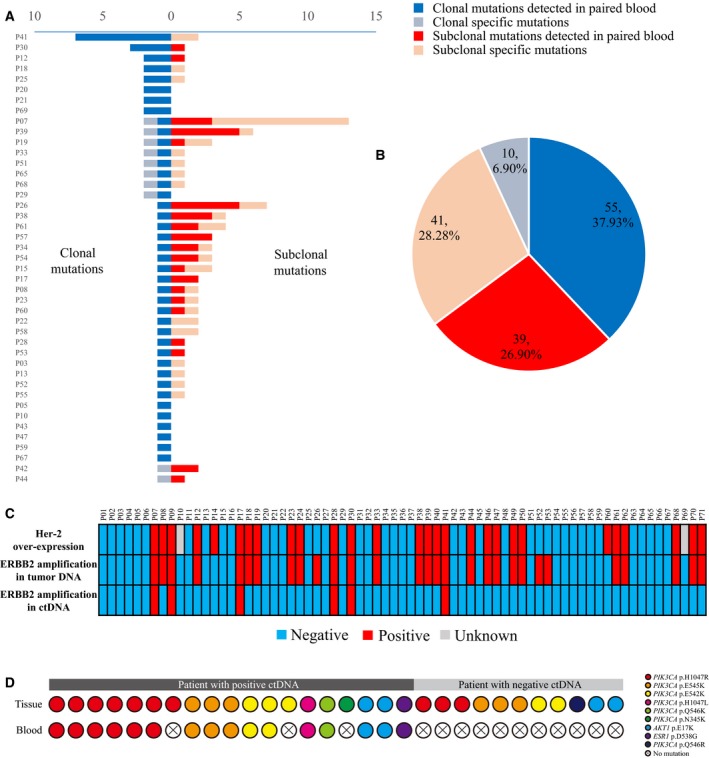
Concordance between genomic alterations in tumor DNA and ctDNA. (A) Distribution of tumor‐derived mutations in each patient. Clonality analysis was performed using the PyClone strategy. (B) Diagram illustrating the overall proportion of clonal and subclonal mutations in tumor tissues. (C) Concordance between detectable CNVs of *ERBB2* and *HER2* overexpression tested by immunohistochemistry. (D) Overview of clinically actionable SNVs between matched tissue and blood samples.

Subsequently, we performed clonality analysis using the PyClone strategy to identify somatic variations in tumor DNA of the 43 patients with tumor‐derived mutations detected in blood. We also investigated differences in the detection rates of tumor‐derived mutations in blood between clonal and subclonal clusters, which revealed collectively 65 clonal mutations (median = 1; range, 1–7) and 80 subclonal mutations in tumor DNA from 33 patients (median = 2; range, 1–13; Fig. 5A). Clonal and subclonal mutations were present in the ctDNA from 41 patients (95.35%) and 20 patients (46.51%), respectively (Fig. 5A). Up to 84.62% of clonal mutations could be detected in matched ctDNA, while only 48.75% of subclonal mutations were found in corresponding control samples (Fig. 5B), further indicating that ctDNA sequencing can capture the majority of tumor‐derived clonal mutations.

### 3.5 Consistency of clinically relevant alterations between tumor DNA and ctDNA

HER2 overexpression that mostly results from CNVs of *ERBB2* notably impacts treatment choice for patients with PBC. To determine the utility of NGS in HER2 status evaluation, we assessed the correlation between HER2 expression measured by immunohistochemistry and *ERBB2* amplification estimated by NGS. Clinically, 28 patients were diagnosed with HER2 overexpression, of whom 26 (92.86%) had detectable *ERBB2* amplification in their tumor DNA. Another three patients with *ERBB2* amplification had normal HER2 status. Excluding two patients with ambiguous HER2 statuses, the overall consistency between these parameters was 92.75%. However, only six (21.43%) patients with HER2 overexpression had detectable *ERBB2* amplification in ctDNA with an overall consistency of 68.12%. Although the positive predictive value of ctDNA detection was 100%, the low negative predictive value (65.08%) limits the clinical application of CNV detection in ctDNA (Fig. 5C).

We next evaluated other clinically actionable mutations. Overall, 30 SNVs in 30 patients were highlighted, including 25 *PIK3CA* coding region variations related with anti‐HER2 regimen resistance and/or mTOR inhibitor sensitivity (Elkabets *et al*., 2013; Majewski *et al*., 2015), four *AKT1* E17K variations targeted by AKT inhibitors (Beaver *et al*., 2013), and one *ESR1* D538G variation inducing resistance to aromatase inhibitors (Wang *et al*., 2016; Fig. 5D). Among 19 patients with positive ctDNA, 16 (84.21%) demonstrated concordantly actionable SNVs in tissue and blood (Fig. 5D).

## 4 Discussion

In recent years, several studies have demonstrated that ctDNA sequencing results correlate with tumor burden and can predict tumor prognosis in patients with breast cancer (Bettegowda *et al*., 2014; Garcia‐Murillas *et al*., 2015; Olsson *et al*., 2015). However, most of these studies have only focused on patients who had undergone multiline treatments or whose cancer had metastasized, while research related to initially diagnosed PBC patients is still scarce. Here, we studied a large cohort of Chinese, initially diagnosed PBC patients to systematically assess the feasibility and reliability of ctDNA genomic profiling to inform tumor management. Results revealed that a patient's ctDNA mutational profile strongly correlated with that of tumor DNA, while some clinicopathological factors affected the detection of tumor‐derived mutations in blood. Based on our findings, ctDNA profiling is a promising method for determining the genomic landscape and potentially best clinical management plan of PBC patients, especially those with advanced‐stage cancer or positive HR status.

In clinical practice, the most common tumor markers utilized in breast cancer diagnosis and prognosis are CA15‐3 and CEA (Martelotto *et al*., 2014; Molina *et al*., 2010; Park *et al*., 2008). However, a study of Chinese breast cancer patients indicates that CEA and CA15‐3 levels are elevated in only 7.2% and 12.3% of these patients, respectively (Garcia‐Murillas *et al*., 2015). Thus, these common biomarkers may not be as reliable or sensitive in Chinese PBC patients compared with other populations. Alternatively, this study revealed that the overall sensitivity of tumor‐derived mutation detection in blood was 67.61%, while the sensitivity in 64 patients with local lesions was 57.81%. Together, these data indicate that ctDNA detection is a feasible complementary biomarker for both early‐ and advanced‐stage PBC patients.

In our study cohort, most of mutated genes in either tumor DNA or ctDNA, including *TP53* and *PIK3CA,* both of which play important roles in tumorigenesis and progression (Muller and Vousden, 2013; Oren and Rotter, 2010; Silwal‐Pandit *et al*., 2014; Zardavas *et al*., 2014), presented higher mutation frequencies than those reported in TCGA. This discrepancy may be partially due to the greater sensitivity of mutation detection for the panel‐based NGS approach used in our study as compared to genomewide sequencing generally used for samples in TCGA. Recently, a study applying PCR‐based sequencing drew similar conclusions (Cohen *et al*., 2018), verifying that advances in sequencing technology could optimize the genomic profiling of tumors.

Because tissue sequencing is still the ‘gold standard’ for molecular profiling of solid tumors, we evaluated the overall concordance of somatic alterations between tumor DNA and ctDNA. While a prior study demonstrated unsatisfactory correspondence, two limitations may have influenced the accuracy of these results: The median interval between the collection of tumor tissues and peripheral blood was 146 days, and ctDNA and tumor DNA were each sequenced using different platforms (Guardant360 and Foundation One) (Chae *et al*., 2017). Alternatively, Kaisaki *et al*. analyzed genomic alterations of ctDNA and primary tumors in 21 melanoma patients and showed an ideal concordance between ctDNA and tumor DNA when samples were collected before treatment, confirming that the interval between sample of tissue and peripheral blood affects sequencing results (Kaisaki *et al*., 2016). In our study, the paired samples of tumor tissue and peripheral blood were collected within 4 days prior to any treatment, and all sequencing procedures were executed uniformly to ensure the reliability and sensitivity of detection. Although gene frequencies showed high concordance between tumor DNA and ctDNA, some differences persisted, such as the high mutation rate of *DNMT3A* in ctDNA. The most likely explanation for this discrepancy is that *DNMT3A* mutations detected in ctDNA are involved in hematopoietic clones with undefined clinical implications, rather than tumor‐derived (Xie *et al*., 2014). In addition to *DNMT3A*, mutations in genes such as *JAK2* and *TET2*, which are also located in hematopoietic clones, can confound the detection of tumor‐derived variants in blood (Xie *et al*., 2014). However, this limitation of ctDNA sequencing can be partially resolved by filtering hematopoietic variations from blood monocyte sequencing results, which we applied in our sequencing strategy. The construction of large‐scale databases encompassing numerous ctDNA sequencing results could further address this issue. Moreover, due to intratumor heterogeneity, single‐site tissue biopsies may lead to incomplete genetic profiling, which could also explain the absence of ctDNA mutations in matched tumor DNA (Cai *et al*., 2017; De Mattos‐Arruda *et al*., 2014).

Based on GO and KEGG analyses, the genetic functions possibly affected by tissue‐specific and overlapping mutations presented similar profiles, and both potentially induced changes in breast cancer‐related pathways, although the enrichment profiles of blood‐specific mutations differed. Molecular biomarkers related to drug sensitivity and resistance were detected in blood, suggesting that tumor‐derived variations in blood may be more clinically relevant than those that are blood‐specific. Although many articles have verified the clinical applicability of tumor‐derived variations in diverse tumor types (Abbosh *et al*., 2017; Esposito *et al*., 2014; Ulz *et al*., 2017), our study is the first to confirm the utility of tumor‐derived variations in blood using basic sequencing analysis. We predict that additional biological and clinical implications of ctDNA profiling will be revealed in further studies with larger populations.

A previous study reported that the detectable rate of mutant ctDNA significantly differs between patients with metastasis (stage IV/M1) and those with localized lesions (stages I–III/M0) (Bettegowda *et al*., 2014). Similarly, in our study, almost all stage IV/M1 patients (85.71%) carried tumor‐derived mutations in blood, whereas the proportions were only 57.81% for patients with stages I–III/M0. We further verified that nodal (N stage) and HR status could independently predict the detection of tumor‐derived variations in blood. Such findings can explain the absence of ctDNA for some patients and help improve the efficiency of ctDNA profiling. Subsequently, we found that the MVAF of ctDNA was significantly higher in patients with metastasis, as well as advanced TNM status, indicating that ctDNA quantity may correlate with clinical tumor load. Longitudinal monitoring of ctDNA is a possible strategy for therapeutic evaluation and tumor surveillance. In addition, ctDNA tracking has been used to assess the effects of surgery and monitor the dynamic changes in tumor load during medical treatment of breast cancer (Garcia‐Murillas *et al*., 2015; Olsson *et al*., 2015), although more efforts are needed to further confirm the reliability and full clinical benefit of ctDNA characterization.

Clonal evolution is an intrinsic process during tumorigenesis and progression, and clonal ‘trunk’ variations that emerge relatively early contain more driver events than subclonal branch variations. Thus, clonal variations may exert more important clinical implications (McGranahan *et al*., 2015). Based on our study, cfDNA could be mostly comprised of tumor‐derived clonal mutations, while the subclonal overlap between matched tissue and blood is limited. Indeed, the incomplete concordance of mutation spectrum between matched tissue and blood is the greatest barrier to the clinical approval of ctDNA (Merker *et al*., 2018). As clonal mutations present superior allele frequencies than subclonal mutations (McGranahan *et al*., 2015), we conclude that sequencing depth for ctDNA may be insufficient to detect whole subclonal mutations. The anatomical site of tumor cell clones should also be considered, as tissue comprised of cellular clones with fewer vessels may be less likely to release mutant fragments into blood circulation.

The application of anti‐HER2 agents has improved the prognosis for PBC patients. Our results revealed that *ERBB2* gene amplification highly correlated with clinical HER2 overexpression, facilitating the decision to administer anti‐HER2 therapy via precise genomic profiling. We also found that some patients carried clinically actionable SNVs in *PIK3CA*,* AKT1*, and *ESR1*. For patients with positive ctDNA, these SNVs could be traced to blood with high probability, indicating that liquid biopsy is potentially useful in medical management of PBC.

In conclusion, our findings highlight the feasibility of ctDNA profiling for genetic analysis of PBC patients. ctDNA detection can be applied in an ideal proportion of patients, and tumor‐derived functional mutations can be traced in ctDNA. Some clinicopathological factors can affect the detectability of ctDNA; thus, we should carefully determine the applicable population and appropriate biomarkers before clinical application of this approach. Some limitations persist. First, the study population is small, and larger‐scale study using uniform sequencing and data analysis could further validate our results. Second, the sensitivity of CNV detection by ctDNA is unsatisfactory, although this issue could be resolved by optimizing analytical procedures. Third, the lack of practical cases involving ctDNA‐based clinical management restricts the translational interpretation of this study. However, our results are still beneficial in promoting a standardized sequencing process and rigorous strategy for identifying PBC‐related mutations. This approach could contribute to improved treatments and outcomes for PBC patients and demonstrates the potential utility of liquid biopsies in tumor surveillance and directed therapy. A subsequent study based on these current findings to strengthen the translational potential of our work is already in progress.

## 5 Conclusion

Mutant ctDNA fragments were detected in the majority of PBC patients, most of which corresponded to tumor‐derived functional mutations, indicating the potential diagnostic value of ctDNA. Multiple clinical and biological factors, including TNM stage, molecular subtypes, and tumor clonality, can affect the detectability of tumor‐derived biomarkers in blood ctDNA and should be considered when comparing mutations across sample sources for prognostic purposes.

## Conflict of interest

The authors declare no conflict of interest.

## Author contributions

YZ and YX are the main experimental designers and writers; CW, RY, and LP have assisted the management of patients and collection of samples; YG, YZ, YL, and JW helped data arrangement and analysis; YG, XX, LY, and XY offered constructive suggestion for study design and analytical methods.

## Supporting information


**Fig. S1**. Diagram illustrating the distribution of lengths of all cfDNA fragments.
**Fig. S2**. Sequencing depth and fraction of coverage over captured regions.
**Fig. S3**. Prevalence of all mutated genes in tumor DNA and ctDNA.
**Fig. S4**. GO analysis for tissue‐specific, blood‐specific, and overlapping mutations.
**Fig. S5**. Comparative analyses of ctDNA maximal VAF between different groups.
**Table S1**. Sequencing panel design.
**Table S2**. Quality control information of sequencing.
