# Supplementary material for: Clinical factors associated with circulating tumor DNA (ctDNA) in primary breast cancer
Source: Mol Oncol. 2019 Feb 6;13(5):1033–46. doi: 10.1002/1878-0261.12456 (PMC6487710; doi:10.1002/1878-0261.12456)
Supplement: Supplementary file 1 — Fig. S1. Diagram illustrating the distribution of lengths of all cfDNA fragments. Fig. S2. Sequencing depth and fraction of coverage over captured regions. Fig. S3. Prevalence of all mutated genes in tumor DNA and ctDNA. Fig. S4. GO analysis for tissue‐specific, blood‐specific, and overlapping mutations. Fig. S5. Comparative analyses of ctDNA maximal VAF between different groups. Table S1. Sequencing panel design. Table S2. Quality control information of sequencing. [file MOL2-13-1033-s001.pdf]

**Supplemental Figures**

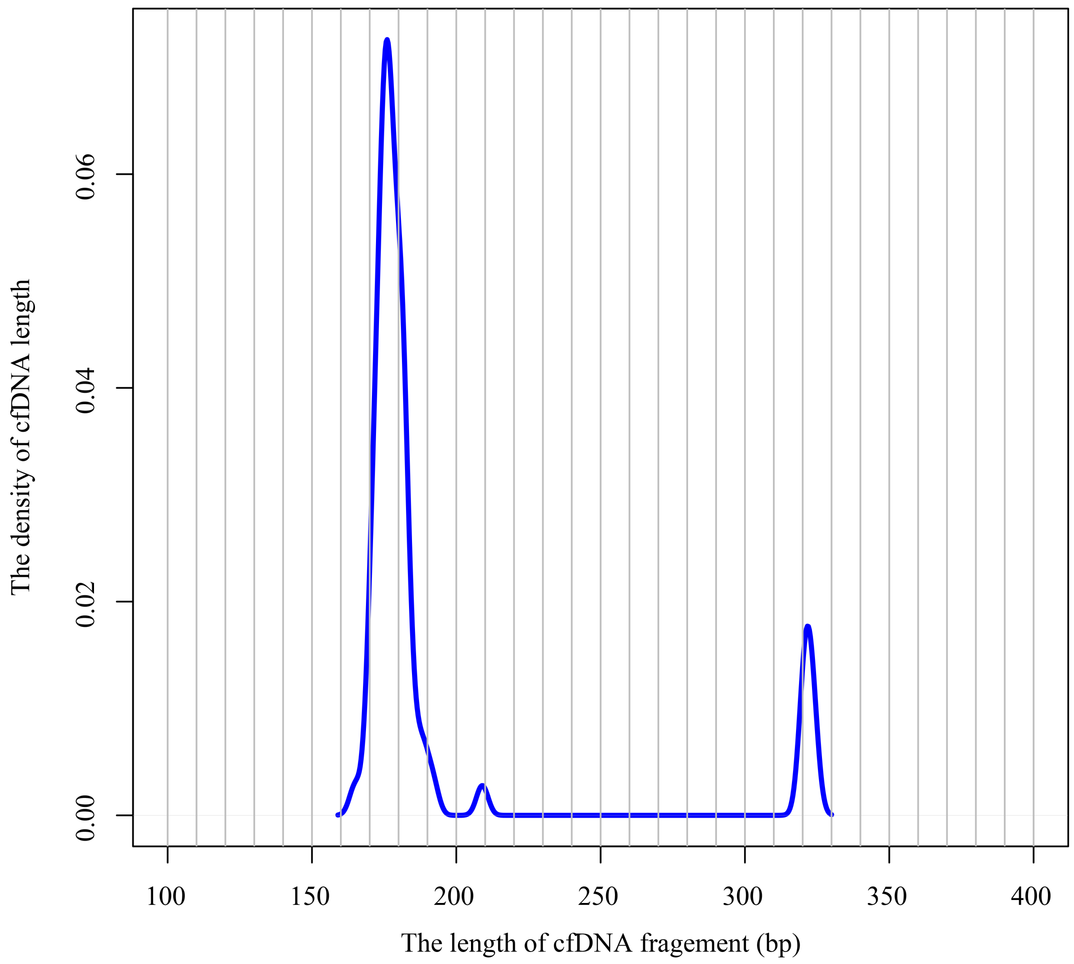


**Fig. S1. Diagram illustrating the distribution of lengths of all cfDNA fragments.** The overall density under the curve was defined as 1.

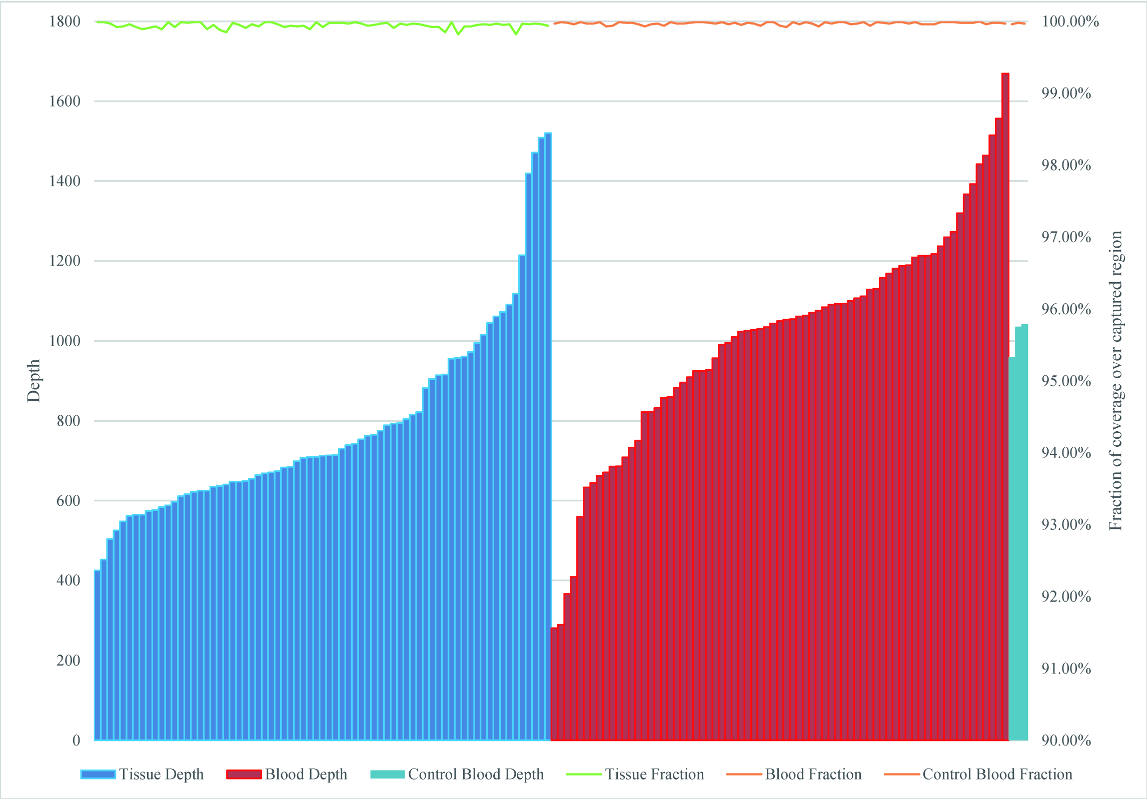


**Fig. S2. Sequencing depth and fraction of coverage over captured regions.** Each column represents the sequencing depth of a tissue or blood sample, while the fraction of each sample is demonstrated with a broken line.

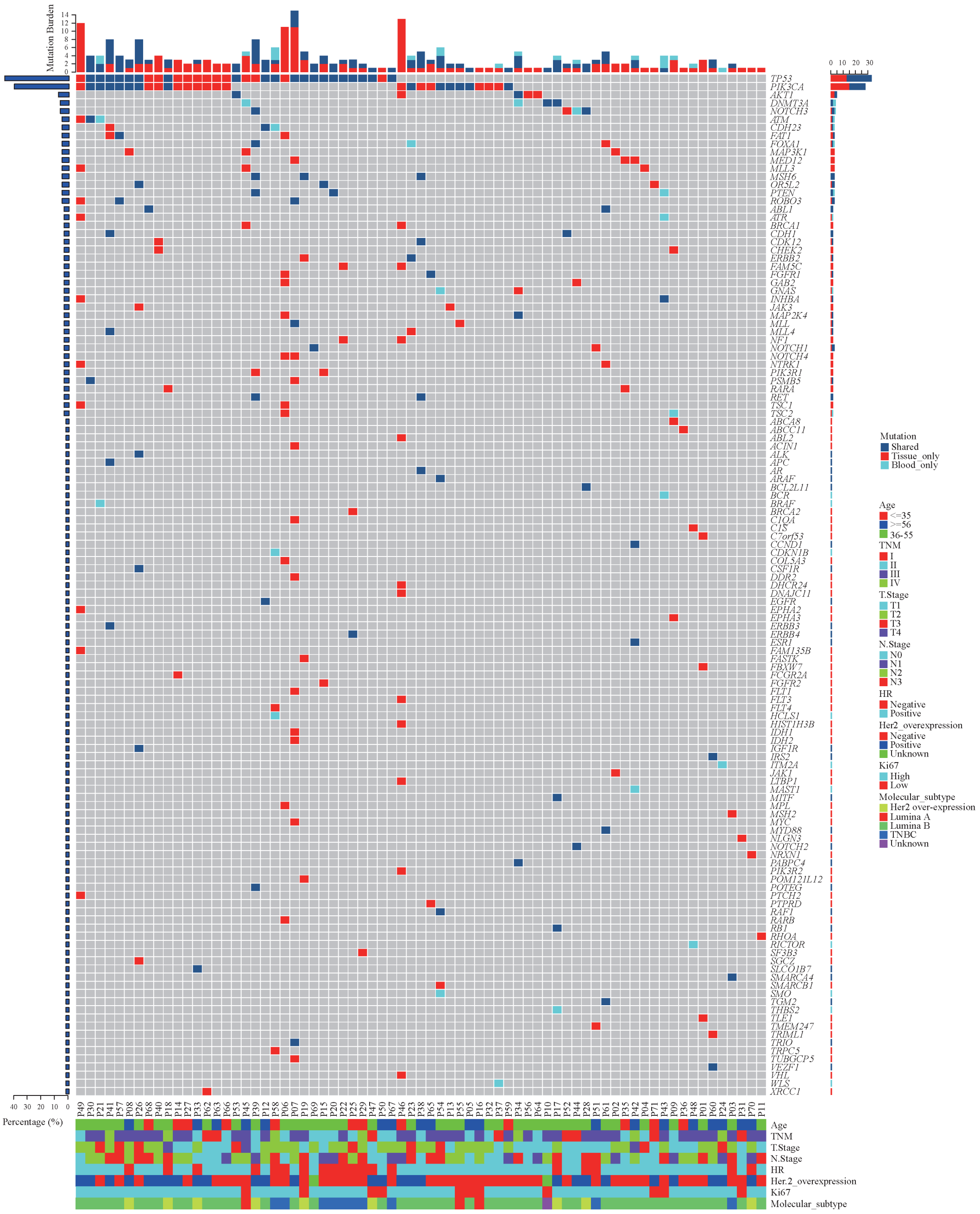


**Fig. S3. Prevalence of all mutated genes in tumor DNA and ctDNA.** The bar chart above indicates the number of genes altered in each patient. The chart below shows the clinical features of patients, including age, tumor, node, metastasis (TNM) stage, T stage, N stage, hormone receptor (HR) overexpression, Her2 status, Ki67 level, and molecular subtypes. The right bar represents the frequency of specific altered genes in the total cohort. Blue: genes

altered in both tumor DNA and ctDNA; red: genes altered in tumor DNA but not paired ctDNA; green: genes altered in ctDNA but not paired tumor DNA; gray: no altered genes detected.

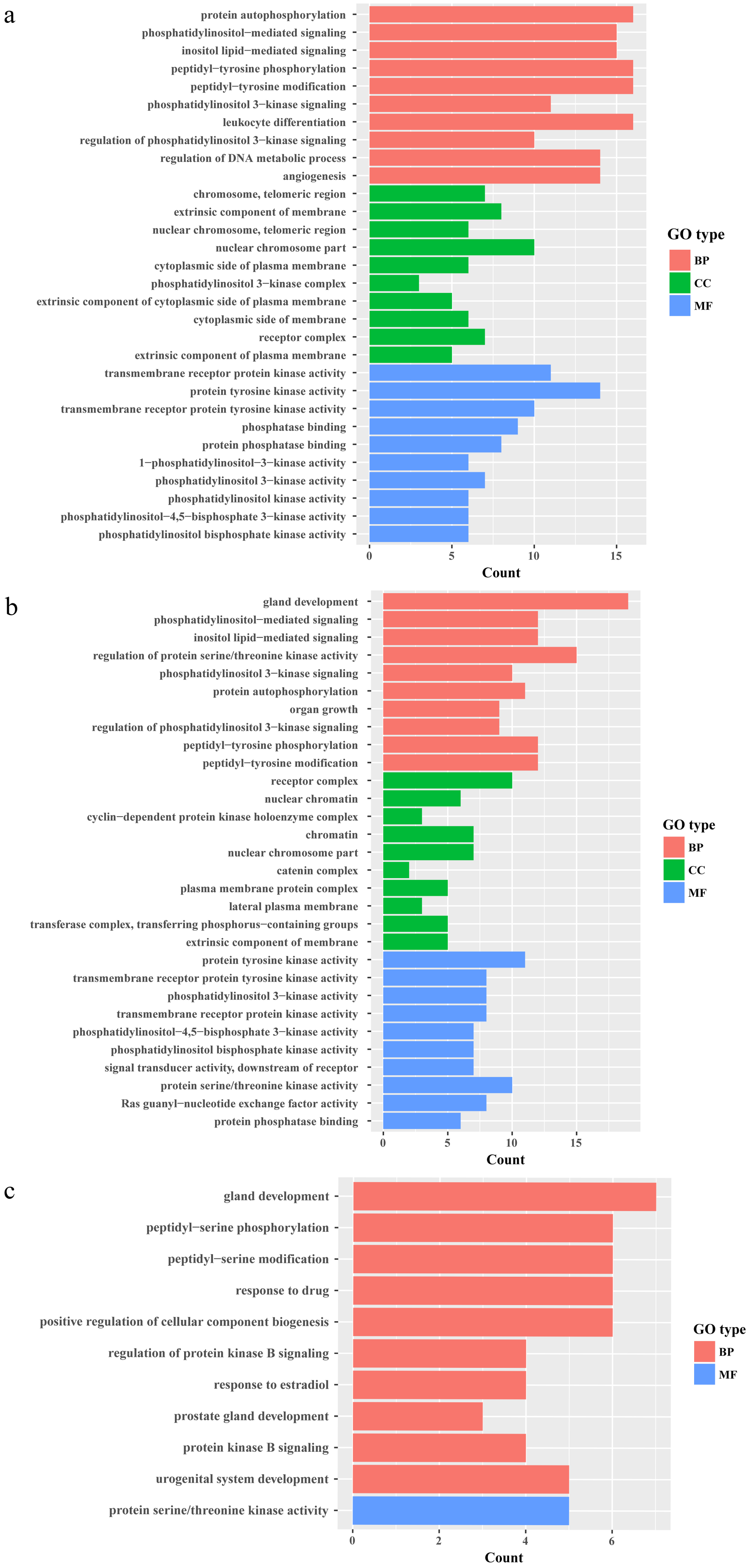
a
protein autophosphorylation
phosphatidylinositol−mediated signaling
inositol lipid−mediated signaling
peptidyl−tyrosine phosphorylation
peptidyl−tyrosine modification
phosphatidylinositol 3−kinase signaling
leukocyte differentiation
regulation of phosphatidylinositol 3−kinase signaling
regulation of DNA metabolic process
angiogenesis
chromosome, telomeric region
extrinsic component of membrane
nuclear chromosome, telomeric region
nuclear chromosome part
cytoplasmic side of plasma membrane
phosphatidylinositol 3−kinase complex
extrinsic component of cytoplasmic side of plasma membrane
cytoplasmic side of membrane
receptor complex
extrinsic component of plasma membrane
transmembrane receptor protein kinase activity
protein tyrosine kinase activity
transmembrane receptor protein tyrosine kinase activity
phosphatase binding
protein phosphatase binding
1−phosphatidylinositol−3−kinase activity
phosphatidylinositol 3−kinase activity
phosphatidylinositol kinase activity
phosphatidylinositol−4,5−bisphosphate 3−kinase activity
phosphatidylinositol bisphosphate kinase activity
0
5
10
15
Count
GO type
BP
CC
MF
b
gland development
phosphatidylinositol−mediated signaling
inositol lipid−mediated signaling
regulation of protein serine/threonine kinase activity
phosphatidylinositol 3−kinase signaling
protein autophosphorylation
organ growth
regulation of phosphatidylinositol 3−kinase signaling
peptidyl−tyrosine phosphorylation
peptidyl−tyrosine modification
receptor complex
nuclear chromatin
cyclin−dependent protein kinase holoenzyme complex
chromatin
nuclear chromosome part
catenin complex
plasma membrane protein complex
lateral plasma membrane
transferase complex, transferring phosphorus−containing groups
extrinsic component of membrane
protein tyrosine kinase activity
transmembrane receptor protein tyrosine kinase activity
phosphatidylinositol 3−kinase activity
transmembrane receptor protein kinase activity
phosphatidylinositol−4,5−bisphosphate 3−kinase activity
phosphatidylinositol bisphosphate kinase activity
signal transducer activity, downstream of receptor
protein serine/threonine kinase activity
Ras guanyl−nucleotide exchange factor activity
protein phosphatase binding
0
5
10
15
Count
GO type
BP
CC
MF
c
gland development
peptidyl−serine phosphorylation
peptidyl−serine modification
response to drug
positive regulation of cellular component biogenesis
regulation of protein kinase B signaling
response to estradiol
prostate gland development
protein kinase B signaling
urogenital system development
protein serine/threonine kinase activity
0
2
4
6
Count
GO type
BP
MF

**Fig. S4. Gene ontology (GO) analysis for tissue-specific, blood-specific, and overlapping mutations.** a) GO analysis for mutated genes with tissue-specific alterations. b) GO analysis for mutated genes with overlapping alterations. c) GO analysis for mutated genes with blood-specific alterations. The length of each column represents the number of enriched genes, and genes are ranked according to statistical significance. BP, biological process; CC, cellular component; MF, molecular function.

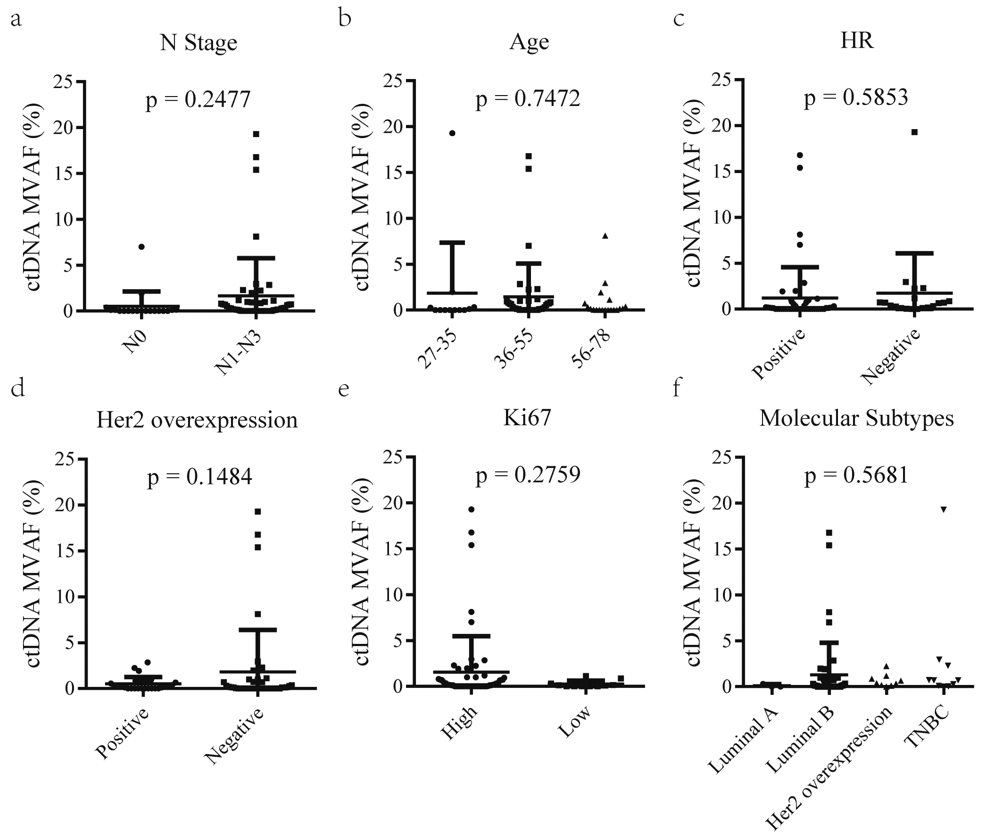


**Fig. S5. Comparative analyses of ctDNA maximal variant allele frequency (MVAF) between different groups.** a) The comparative analysis of ctDNA MVAF in patients with different N stages. b) The comparative analysis of ctDNA MVAF in various diagnostic age groups. c) The comparative analysis of ctDNA MVAF in groups with various hormone receptor (HR) statuses. d) The comparative analysis of ctDNA MVAF between the patients with various Her2 statuses. e) The comparative analysis of ctDNA MVAF between the patients with various Ki67 levels. f) The comparative analysis of ctDNA MVAF between the patients with various molecular subtypes.

## Supplemental Tables

**Table S1. Sequencing panel design.**

| | | | | |
|---|---|---|---|---|
| **Genes with all exons captured (170 genes, 2735 exons)** | | | | |
| *ABL1* | *CDK4* | *FGFR4* | *MDM4* | *PSMB1* |
| *ABL2* | *CDK6* | *FLCN* | *MED12* | *PSMB5* |
| *AKT1* | *CDK8* | *FLT1* | *MET* | *PTCH1* |
| *AKT2* | *CDKN1A* | *FLT3* | *MITF* | *PTCH2* |
| *AKT3* | *CDKN1B* | *FLT4* | *MLH1* | *PTEN* |
| *ALK* | *CDKN2A* | *FOXA1* | *MLH3* | *PTPN11* |
| *APC* | *CDKN2B* | *FOXL2* | *MPL* | *RAF1* |
| *AR* | *CHEK1* | *GAB2* | *MS4A1* | *RARA* |
| *ARAF* | *CHEK2* | *GATA3* | *MSH2* | *RB1* |
| *ATM* | *CRKL* | *GNA11* | *MSH3* | *RET* |
| *ATR* | *CSF1R* | *GNAQ* | *MSH6* | *RHEB* |
| *AURKA* | *CTNNB1* | *GNAS* | *MTOR* | *RHOA* |
| *AURKB* | *DDR1* | *HDAC1* | *MYC* | *RICTOR* |
| *AXL* | *DDR2* | *HDAC4* | *MYD88* | *RNF43* |
| *BAP1* | *DNMT3A* | *HGF* | *NF1* | *ROCK1* |
| *BCL2* | *EGFR* | *HRAS* | *NF2* | *ROS1* |
| *BRAF* | *EPHA2* | *IDH1* | *NOTCH1* | *RPS6KB1* |
| *BRCA1* | *EPHA3* | *IDH2* | *NOTCH2* | *SMARCA4* |
| *BRCA2* | *EPHA5* | *IGF1R* | *NOTCH3* | *SMARCB1* |
| *BRD2* | *ERBB2* | *IL7R* | *NOTCH4* | *SMO* |
| *BRD3* | *ERBB3* | *INPP4B* | *NRAS* | *SRC* |
| *BRD4* | *ERBB4* | *IRS2* | *NTRK1* | *STAT1* |
| *BTK* | *ERCC1* | *JAK1* | *NTRK3* | *STAT3* |
| *C11orf30* | *ERG* | *JAK2* | *PALB2* | *STK11* |
| *C1QA* | *ESR1* | *JAK3* | *PDGFRA* | *SYK* |
| *C1S* | *EZH2* | *KDR* | *PDGFRB* | *TMPRSS2* |
| *CBL* | *FAT1* | *KIT* | *PDK1* | *TOP1* |
| *CCND1* | *FBXW7* | *KRAS* | *PIK3CA* | *TP53* |
| *CCND2* | *FCGR2A* | *MAP2K1* | *PIK3CB* | *TSC1* |
| *CCND3* | *FCGR2B* | *MAP2K2* | *PIK3R1* | *TSC2* |
| *CCNE1* | *FCGR3A* | *MAPK1* | *PIK3R2* | *VEGFA* |
| *CD274* | *FGFR1* | *MAPK3* | *PMS1* | *VHL* |
| *CDH1* | *FGFR2* | *MCL1* | *PMS2* | *XPO1* |
| *CDK13* | *FGFR3* | *MDM2* | *PRKAA1* | *XRCC1* |
| **Genes with partial exons captured (847 genes, 1122 exons)** | | | | |
| *ABCA10* | *ABCA8* | *ABCB7* | *ABCC8* | *ABCF2* |
| *ACE* | *ACER2* | *ACOT11* | *ACPP* | *ACSL1* |
| *ACSM5* | *ACSS3* | *ACTL6B* | *ADAM23* | *ADAM33* |
| *ADAMTS12* | *ADAMTS16* | *ADAMTS19* | *ADAMTS20* | *ADAMTS5* |
| *ADAMTSL1* | *ADD2* | *AGMAT* | *AGTPBP1* | *AHCTF1* |
| *AK5* | *AKR1B10* | *AKR1C1* | *ALDH1A3* | *ALDH2* |
| *ALG5* | *ALX4* | *AMOT* | *ANK2* | *ANKRD13D* |
| *ANKRD20A4* | *ANKRD27* | *ANKRD28* | *ANKRD30A* | *ANKRD30B* |
| *ANKRD36B* | *ANO2* | *AP1B1* | *AP1G2* | *AP3B1* |
| *APAF1* | *APLP2* | *APMAP* | *APPL2* | *AQP12A* |
| *ARFGAP1* | *ARFRP1* | *ARHGAP35* | *ARHGAP40* | *ARHGEF1* |
| *ARHGEF7* | *ARNTL* | *ARPC4-TTLL3* | *ASH2L* | *ASTN1* |
| *ASXL2* | *ATAD2B* | *ATG9B* | *ATP10B* | *ATP10D* |

| | | | | |
|---|---|---|---|---|
| *ATP12A* | *ATP2C1* | *ATP6V0A2* | *ATP8B2* | *ATXN2* |
| *ATXN7L2* | *BAX* | *BBS9* | *BCAS1* | *BCAS2* |
| *BLOC1S1* | *BMPR1B* | *BRF1* | *BRSK2* | *BRWD3* |
| *BSG* | *BTNL3* | *BTRC* | *C12orf5* | *C19orf38* |
| *C1orf112* | *C1orf35* | *C20orf112* | *C2orf47* | *C2orf62* |
| *C7orf53* | *C9orf114* | *C9orf43* | *CACNA1A* | *CACNA1D* |
| *CACNA1E* | *CADM2* | *CAMKK1* | *CAPRIN1* | *CARS* |
| *CARS2* | *CASC4* | *CASP8* | *CASP8AP2* | *CASQ2* |
| *CATSPER2* | *CBFB* | *CBX4* | *CCDC155* | *CCDC159* |
| *CCDC17* | *CCT3* | *CCT6B* | *CD1E* | *CD300LF* |
| *CD5L* | *CD9* | *CD97* | *CD99* | *CDH18* |
| *CDH24* | *CDH26* | *CDK11A* | *CDK12* | *CDK14* |
| *CDK18* | *CDK19* | *CDS1* | *CEACAM20* | *CECR2* |
| *CELA2B* | *CGN* | *CHD3* | *CHD4* | *CHD6* |
| *CHI3L1* | *CISD3* | *CLCN7* | *CLEC16A* | *CLINT1* |
| *CNGB3* | *CNKSR2* | *CNOT3* | *CNOT4* | *CNTN1* |
| *CNTN4* | *CNTN5* | *CNTNAP3B* | *CNTNAP5* | *COASY* |
| *COL14A1* | *COL16A1* | *COL19A1* | *COL1A1* | *COL25A1* |
| *COL4A5* | *COL4A6* | *COL5A1* | *COL5A2* | *COL5A3* |
| *COL6A5* | *COL6A6* | *COL9A1* | *COPA* | *COPG1* |
| *CPA1* | *CPSF3* | *CPSF6* | *CRTAM* | *CRTAP* |
| *CRYBG3* | *CSMD1* | *CSMD3* | *CSN3* | *CSNK1E* |
| *CSPP1* | *CTCF* | *CTIF* | *CTNNA2* | *CTSF* |
| *CYP2A13* | *CYP3A4* | *CYP4A11* | *CYTH4* | *DCLK2* |
| *DCST1* | *DDB1* | *DDX24* | *DDX3X* | *DEPDC4* |
| *DGKK* | *DHCR24* | *DHDDS* | *DHX9* | *DIAPH1* |
| *DKC1* | *DLST* | *DMD* | *DMXL1* | *DMXL2* |
| *DNAH10* | *DNAH5* | *DNAH9* | *DNAJC11* | *DNAJC9* |
| *DNTTIP1* | *DOCK11* | *DOCK3* | *DOT1L* | *DPP10* |
| *DPP4* | *DRGX* | *DUOX1* | *DYSF* | *DZANK1* |
| *ECHDC1* | *EDN1* | *EEF1A1* | *EFCAB5* | *EFCAB6* |
| *EFCAB7* | *EFHA2* | *EFNA5* | *EIF1AX* | *EIF2B5* |
| *EIF2C2* | *EIF3E* | *EIF3I* | *EIF4ENIF1* | *EIF4H* |
| *ELAVL3* | *ELL3* | *EMID2* | *ENPP2* | *ENTPD6* |
| *EPB41L2* | *EPB41L4B* | *EPHB1* | *EPS8L3* | *ESD* |
| *ETNK2* | *ETV6* | *EXOC4* | *EXOC5* | *EXOC6* |
| *EXOC7* | *EXTL3* | *EYA4* | *F8* | *F9* |
| *FAH* | *FAM114A2* | *FAM131B* | *FAM135B* | *FAM13C* |
| *FAM157B* | *FAM177B* | *FAM21A* | *FAM3A* | *FAM49A* |
| *FAM49B* | *FAM5C* | *FAM86B1* | *FAN1* | *FANCC* |
| *FASTK* | *FATE1* | *FBN2* | *FDCSP* | *FLNC* |
| *FLOT2* | *FLT3LG* | *FMN2* | *FMNL3* | *FNDC4* |
| *FNIP2* | *FOLH1* | *FOXJ2* | *FRG1* | *FRG2B* |
| *FRMD4A* | *FRMPD2* | *FRMPD4* | *FSD2* | *FSHR* |
| *FUBP1* | *FUNDC1* | *GAB3* | *GABRD* | *GAD2* |
| *GALNT13* | *GALNT14* | *GFRAL* | *GIGYF1* | *GINS4* |
| *GIPR* | *GKN2* | *GLB1L3* | *GLYR1* | *GMDS* |
| *GNPTAB* | *GOLGA4* | *GPAT2* | *GPATCH2* | *GPR114* |
| *GPR125* | *GPR133* | *GPR144* | *GPS2* | *GRIA3* |
| *GRIK2* | *GUCY1A3* | *GUCY2C* | *GYLTL1B* | *HAAO* |
| *HAP1* | *HAUS5* | *HAUS6* | *HCN1* | *HDAC6* |
| *HEATR7B2* | *HECTD4* | *HECW1* | *HECW2* | *HID1* |
| *HIST1H3B* | *HLA-DRB1* | *HLA-DRB5* | *HMCN1* | *HMHA1* |
| *HNF4A* | *HOMER2* | *HPS3* | *HPS4* | *HSPA12B* |

| | | | | |
|---|---|---|---|---|
| *HSPD1* | *HYDIN* | *IBSP* | *IFT172* | *IGSF9* |
| *IKBKAP* | *IKBKE* | *IL11RA* | *IL13RA2* | *IL1RAPL1* |
| *IL27RA* | *IMPG1* | *INHBA* | *INPP5J* | *IQCA1* |
| *ITFG2* | *ITGA8* | *ITGA9* | *ITIH1* | *ITLN2* |
| *ITM2A* | *ITPKB* | *ITPR1* | *KCNAB2* | *KCNH6* |
| *KCNQ2* | *KDM4A* | *KDM6A* | *KEAP1* | *KIAA0195* |
| *KIAA0226* | *KIAA0319* | *KIAA0922* | *KIAA1191* | *KIAA1199* |
| *KIAA1211L* | *KIF13A* | *KIF1B* | *KIF26B* | *KIFAP3* |
| *KIFC1* | *KIR2DL3* | *KIR3DL3* | *KLHL1* | *KLHL14* |
| *KLK1* | *KMT2B* | *KMT2C* | *KRT2* | *KRT9* |
| *KRTAP5-5* | *KTN1* | *L3MBTL1* | *LARP1* | *LCN10* |
| *LCT* | *LCTL* | *LETM1* | *LGALS13* | *LILRB3* |
| *LILRB4* | *LIPN* | *LMAN1L* | *LMBR1L* | *LPCAT4* |
| *LPHN3* | *LRBA* | *LRP1B* | *LRP2* | *LRP4* |
| *LRRC16B* | *LRRC2* | *LRRC7* | *LRRC72* | *LRRD1* |
| *LRRFIP2* | *LRSAM1* | *LTBP1* | *LUC7L2* | *LUZP4* |
| *MAEL* | *MAGI1* | *MAN2A1* | *MAP2* | *MAP2K4* |
| *MAP3K1* | *MAP4K1* | *MAPKAPK3* | *MAPRE3* | *MAST1* |
| *MBIP* | *MBTPS2* | *MCF2L2* | *MCOLN2* | *MDGA2* |
| *MDN1* | *MED23* | *MEFV* | *METTL14* | *METTL5* |
| *MGAM* | *MICALL1* | *MID1* | *MIER2* | *MLL* |
| *MLPH* | *MORC1* | *MORN1* | *MRPL1* | *MRPL24* |
| *MRPS18B* | *MSI1* | *MTA2* | *MTM1* | *MTR* |
| *MTTP* | *MUC5B* | *MUS81* | *MYB* | *MYBPC2* |
| *MYCBP2* | *MYH15* | *MYH2* | *MYH4* | *MYH8* |
| *MYH9* | *MYL5* | *MYL6* | *MYLK2* | *MYO3A* |
| *MYOM1* | *NACAD* | *NARF* | *NAT10* | *NAV3* |
| *NBPF1* | *NBPF10* | *NCF2* | *NCKAP1* | *NCOR1* |
| *NCOR2* | *NEK5* | *NELL1* | *NFE2L2* | *NIPBL* |
| *NLGN3* | *NLRC3* | *NLRP4* | *NMI* | *NOP2* |
| *NOS1* | *NOS2* | *NRXN1* | *NRXN2* | *NT5C3L* |
| *NTM* | *NUDCD2* | *NUP205* | *NUP210* | *NUTM1* |
| *NWD1* | *NXF1* | *NXF5* | *OBP2A* | *OBP2B* |
| *OCA2* | *ODZ3* | *OR2T4* | *OR4A15* | *OR4C6* |
| *OR5L2* | *OR6F1* | *OSBPL10* | *OTOA* | *OTOGL* |
| *OVCH1* | *P4HB* | *PABPC4* | *PACS2* | *PAEP* |
| *PAGE1* | *PARK2* | *PARP4* | *PCK2* | *PCLO* |
| *PCNT* | *PCNXL2* | *PCSK5* | *PCYT1A* | *PDCD6* |
| *PDE1C* | *PDE2A* | *PDE4DIP* | *PDIA5* | *PDILT* |
| *PDRG1* | *PEX6* | *PGAP1* | *PHACTR3* | *PHF20L1* |
| *PHYH* | *PI4KB* | *PIP4K2C* | *PIP5K1C* | *PIWIL1* |
| *PKD1L2* | *PKHD1* | *PKLR* | *PLAC8* | *PLCB4* |
| *PLCZ1* | *PLEC* | *PLK2* | *PLOD3* | *PLXNA1* |
| *POLDIP2* | *POLE* | *POLR2J* | *POLR3B* | *POLR3GL* |
| *POLRMT* | *POM121L12* | *POTEG* | *PPA1* | *PPDPF* |
| *PPEF1* | *PPFIBP2* | *PPIL2* | *PPP1R17* | *PPP4R4* |
| *PQBP1* | *PREB* | *PREX2* | *PRKACA* | *PRKAG3* |
| *PRKCD* | *PRKDC* | *PRKX* | *PRRX1* | *PRSS1* |
| *PRUNE* | *PSG2* | *PSG5* | *PSIP1* | *PSMC4* |
| *PSMC6* | *PSTPIP1* | *PTBP3* | *PTCD3* | *PTGES3L-AARSD1* |
| *PTGS2* | *PTPLAD1* | *PTPN13* | *PTPRA* | *PTPRD* |
| *PTPRM* | *PYHIN1* | *QRICH2* | *RAB1B* | *RAB3GAP2* |
| *RAB6A* | *RAC2* | *RALBP1* | *RAPGEF2* | *RARB* |

| | | | | |
|---|---|---|---|---|
| RASEF | RBM6 | RBMX | RCC1 | REC8 |
| REG1B | RELN | RERE | RFWD2 | RFX3 |
| RNF215 | RNF219 | RPL22 | RPL36A | RPS5 |
| RPS6KA1 | RPTOR | RPUSD4 | RREB1 | RRP7A |
| RUNDC3A | RUNX1 | RYR2 | RYR3 | SAFB2 |
| SAG | SAGE1 | SAMD8 | SCN10A | SCN3A |
| SCN7A | SCN9A | SDK2 | SEC14L4 | SEC24B |
| SEH1L | SELP | SEMA6A | SEPT12. | SERPINA7 |
| SETD1B | SETD2 | SF1 | SF3B1 | SF3B14 |
| SF3B3 | SGCZ | SGIP1 | SGK1 | SGPL1 |
| SH2D3A | SH3BGR | SH3PXD2A | SHISA4 | SI |
| SIDT2 | SIK3 | SIM1 | SIM2 | SLC13A3 |
| SLC17A6 | SLC17A8 | SLC25A1 | SLC25A30 | SLC26A3 |
| SLC2A2 | SLC30A5 | SLC35B2 | SLC35B4 | SLC38A4 |
| SLC38A5 | SLC43A1 | SLC45A1 | SLC4A10 | SLC4A4 |
| SLC5A1 | SLC6A5 | SLC8A1 | SLCO1B7 | SLCO5A1 |
| SMTN | SNTG1 | SORCS3 | SPAG16 | SPATA13 |
| SPG20 | SPINT1 | SPPL2A | SPPL3 | SPRED1 |
| SPTA1 | SRRT | SSBP3 | SSH2 | SSPO |
| ST18 | ST6GALNAC1 | STAG2 | STAT4 | STAT6 |
| STK11IP | STK31 | STX3 | SULT1A4 | SUPT5H |
| SUPT6H | SYCP2L | SYNE1 | SYNE2 | SYNJ2 |
| TAF1B | TAF6 | TARBP1 | TBC1D1 | TBC1D21 |
| TBC1D3 | TBC1D5 | TBL1X | TBP | TBX15 |
| TBX22 | TBX3 | TCF20 | TCF4 | TCP10 |
| TCP11 | TEK | TESC | TEX35 | TFDP1 |
| TGDS | TGM2 | TGM5 | THBS2 | THEM5 |
| THOC1 | THSD7A | THSD7B | TIMD4 | TIMM44 |
| TIMP3 | TJP3 | TLE1 | TLL1 | TMC2 |
| TMED8 | TMEM104 | TMEM120B | TMEM132D | TMEM145 |
| TMEM247 | TMEM80 | TMEM87A | TMTC4 | TMX3 |
| TNFAIP6 | TNFSF4 | TNN | TNNT1 | TNR |
| TNS3 | TP53BP1 | TPCN1 | TPH2 | TPMT |
| TPTE | TRIM33 | TRIM51 | TRIM58 | TRIML1 |
| TRIO | TRIP11 | TRMT112 | TRPC5 | TRUB1 |
| TSGA10 | TSKS | TSPAN12 | TSR2 | TTF2 |
| TTN | TUBA3C | TUBGCP4 | TUBGCP5 | TYK2 |
| TYRP1 | U2AF1 | U2AF2 | UBASH3A | UBE2Q1 |
| UBE4B | UCHL3 | UCK2 | UGT8 | ULK3 |
| UMOD | UNC13A | UNC13D | UNC5D | USP12 |
| USP34 | USP39 | USP45 | USP48 | VAV1 |
| VEZF1 | VILL | VIT | VPS13A | VPS33B |
| VSIG4 | WAS | WASL | WDR44 | WDR52 |
| WDR62 | WDR66 | WDR72 | WDTC1 | WLS |
| WSCD2 | WWP2 | XBP1 | XPO4 | XPO5 |
| ZAP70 | ZBTB8OS | ZC3H13 | ZC3H7B | ZDHHC11 |
| ZFC3H1 | ZFR | ZMYM4 | ZNF143 | ZNF350 |
| ZNF385A | ZNF414 | ZNF512B | ZNF541 | ZNF563 |
| ZNF614 | ZNF687 | ZNF705B | ZNF705G | ZNF711 |
| ZNF804B | ZSWIM8 | | | |

36

**Table S2. Quality control information of sequencing.**

| Patient* | Sample | cfDNA (ng/ml) | Average depth (×) | Capture ratio | Mapping rate | Mismatch rate |
|---|---|---|---|---|---|---|
| C01 | Plasma | 4.58 | 1036.12 | 33.28% | 97.86% | 0.76% |
| C02 | Plasma | 4.81 | 1042.27 | 32.42% | 98.08% | 0.85% |
| C03 | Plasma | 5.43 | 960.07 | 32.23% | 97.98% | 0.76% |
| P01 | Plasma | 5.40 | 883.21 | 47.81% | 97.71% | 0.79% |
| P02 | Plasma | 6.70 | 1093.82 | 46.90% | 98.19% | 0.77% |
| P03 | Plasma | 9.75 | 1272.55 | 37.13% | 97.80% | 0.85% |
| P04 | Plasma | 3.90 | 822.64 | 54.36% | 97.45% | 0.78% |
| P05 | Plasma | 11.70 | 1236.83 | 39.32% | 97.20% | 0.81% |
| P06 | Plasma | 3.58 | 823.05 | 21.05% | 97.24% | 0.75% |
| P07 | Plasma | 4.22 | 708.86 | 17.68% | 99.23% | 0.19% |
| P08 | Plasma | 6.60 | 1131.22 | 46.98% | 98.24% | 0.77% |
| P09 | Plasma | 5.60 | 1031.13 | 49.38% | 98.39% | 0.75% |
| P10 | Plasma | 8.26 | 1213.34 | 38.09% | 98.33% | 0.17% |
| P11 | Plasma | 10.00 | 1187.56 | 47.19% | 98.24% | 0.77% |
| P12 | Plasma | 6.30 | 1063.72 | 46.73% | 97.94% | 0.78% |
| P13 | Plasma | 6.34 | 927.41 | 38.87% | 97.83% | 0.84% |
| P14 | Plasma | 5.30 | 957.17 | 43.04% | 97.78% | 0.77% |
| P15 | Plasma | 16.25 | 1112.07 | 34.96% | 97.87% | 0.84% |
| P16 | Plasma | 6.50 | 995.27 | 46.26% | 97.95% | 0.78% |
| P17 | Plasma | 4.97 | 859.47 | 35.44% | 98.15% | 0.16% |
| P18 | Plasma | 5.10 | 1054.7 | 38.29% | 96.79% | 0.80% |
| P19 | Plasma | 5.67 | 409.67 | 34.29% | 98.23% | 0.15% |
| P20 | Plasma | 5.92 | 685.95 | 34.86% | 98.15% | 0.15% |
| P21 | Plasma | 4.32 | 559.43 | 12.99% | 99.20% | 0.19% |
| P22 | Plasma | 4.60 | 1027.62 | 47.14% | 97.93% | 0.79% |
| P23 | Plasma | 3.38 | 662.8 | 35.83% | 97.99% | 0.16% |
| P24 | Plasma | 11.00 | 1259.63 | 47.97% | 98.74% | 0.33% |
| P25 | Plasma | 3.90 | 750.6 | 53.28% | 97.44% | 0.77% |
| P26 | Plasma | 16.98 | 1514.38 | 42.18% | 98.53% | 0.17% |
| P27 | Plasma | 8.50 | 1213.21 | 47.53% | 97.35% | 0.80% |
| P28 | Plasma | 7.02 | 1026.4 | 41.63% | 98.39% | 0.17% |
| P29 | Plasma | 4.60 | 895.85 | 47.60% | 97.64% | 0.80% |
| P30 | Plasma | 5.90 | 1043.8 | 44.55% | 98.85% | 0.34% |
| P31 | Plasma | 8.20 | 1168.67 | 46.06% | 97.66% | 0.80% |
| P32 | Plasma | 8.40 | 1049.7 | 47.80% | 97.99% | 0.78% |
| P33 | Plasma | 8.30 | 1023.42 | 45.29% | 97.41% | 0.81% |
| P34 | Plasma | 19.50 | 1442.36 | 47.90% | 98.87% | 0.33% |
| P35 | Plasma | 3.60 | 857.48 | 54.09% | 97.56% | 0.77% |
| P36 | Plasma | 6.60 | 1091.28 | 47.63% | 98.00% | 0.79% |
| P37 | Plasma | 4.70 | 909.27 | 37.51% | 96.92% | 0.80% |
| P38 | Plasma | 10.27 | 644.5 | 35.40% | 98.50% | 0.14% |
| P39 | Plasma | 2.74 | 632.96 | 32.65% | 98.31% | 0.15% |
| P40 | Plasma | 7.00 | 1076.36 | 49.06% | 98.53% | 0.74% |
| P41 | Plasma | 14.42 | 1367.04 | 27.71% | 99.19% | 0.18% |
| P42 | Plasma | 5.98 | 1557.15 | 40.35% | 97.44% | 0.81% |
| P43 | Plasma | 5.00 | 1053.44 | 49.86% | 98.46% | 0.75% |
| P44 | Plasma | 4.60 | 832.59 | 43.61% | 97.13% | 0.79% |
| P45 | Plasma | 8.50 | 1209.1 | 38.48% | 97.11% | 0.80% |
| P46 | Plasma | 8.40 | 1180.83 | 47.48% | 98.80% | 0.33% |

| | | | | | | |
|---|---|---|---|---|---|---|
| P47 | Plasma | 6.80 | 1129 | 49.29% | 98.66% | 0.75% |
| P48 | Plasma | 8.60 | 1216.91 | 47.39% | 97.89% | 0.78% |
| P49 | Plasma | 8.30 | 1157.53 | 39.20% | 97.38% | 0.80% |
| P50 | Plasma | 8.10 | 1100.51 | 43.53% | 97.64% | 0.77% |
| P51 | Plasma | 20.09 | 1669.07 | 30.16% | 99.52% | 0.20% |
| P52 | Plasma | 8.50 | 1106.71 | 43.31% | 97.94% | 0.77% |
| P53 | Plasma | 15.30 | 1463.9 | 49.63% | 97.80% | 0.78% |
| P54 | Plasma | 9.78 | 1392.81 | 43.31% | 98.56% | 0.17% |
| P55 | Plasma | 4.82 | 366.56 | 11.23% | 99.24% | 0.18% |
| P56 | Plasma | 4.02 | 925.42 | 34.52% | 97.96% | 0.77% |
| P57 | Plasma | 4.54 | 289.59 | 11.14% | 99.28% | 0.18% |
| P58 | Plasma | 2.97 | 280.61 | 10.55% | 99.39% | 0.18% |
| P59 | Plasma | 5.80 | 925.43 | 45.45% | 97.96% | 0.77% |
| P60 | Plasma | 6.00 | 990.64 | 46.03% | 98.10% | 0.77% |
| P61 | Plasma | 6.80 | 1084.55 | 46.84% | 98.23% | 0.76% |
| P62 | Plasma | 5.90 | 1010.11 | 43.74% | 97.69% | 0.77% |
| P63 | Plasma | 12.10 | 1189.13 | 39.57% | 97.20% | 0.81% |
| P64 | Plasma | 7.80 | 1061.74 | 47.70% | 97.44% | 0.79% |
| P65 | Plasma | 2.70 | 670.8 | 53.80% | 97.29% | 0.78% |
| P66 | Plasma | 6.70 | 1071.23 | 44.85% | 97.54% | 0.80% |
| P67 | Plasma | 10.00 | 1319.51 | 48.41% | 97.60% | 0.79% |
| P68 | Plasma | 7.80 | 1093.25 | 46.48% | 98.04% | 0.78% |
| P69 | Plasma | 3.30 | 685.93 | 46.56% | 98.55% | 0.76% |
| P70 | Plasma | 6.00 | 1035.01 | 46.44% | 98.17% | 0.78% |
| P71 | Plasma | 3.20 | 732.88 | 36.89% | 96.69% | 0.80% |
| P01 | Tissue | NA | 712.99 | 49.17% | 99.13% | 0.76% |
| P02 | Tissue | NA | 625.56 | 43.62% | 99.02% | 0.40% |
| P03 | Tissue | NA | 1214.08 | 51.45% | 97.87% | 0.85% |
| P04 | Tissue | NA | 730.19 | 38.70% | 97.86% | 0.89% |
| P05 | Tissue | NA | 576.86 | 31.18% | 97.46% | 0.85% |
| P06 | Tissue | NA | 1091.12 | 49.52% | 98.07% | 0.85% |
| P07 | Tissue | NA | 1519.92 | 51.62% | 98.64% | 0.77% |
| P08 | Tissue | NA | 425.25 | 43.54% | 99.02% | 0.41% |
| P09 | Tissue | NA | 914.17 | 48.15% | 99.13% | 0.77% |
| P10 | Tissue | NA | 503.89 | 35.49% | 98.75% | 0.26% |
| P11 | Tissue | NA | 714.08 | 45.13% | 99.03% | 0.40% |
| P12 | Tissue | NA | 1118.69 | 49.33% | 99.01% | 0.77% |
| P13 | Tissue | NA | 1508.4 | 49.28% | 98.67% | 0.77% |
| P14 | Tissue | NA | 794.21 | 40.92% | 97.87% | 0.88% |
| P15 | Tissue | NA | 994.97 | 49.20% | 98.06% | 0.86% |
| P16 | Tissue | NA | 640.41 | 45.95% | 99.18% | 0.78% |
| P17 | Tissue | NA | 713.6 | 37.98% | 98.77% | 0.26% |
| P18 | Tissue | NA | 763.16 | 46.91% | 97.28% | 0.85% |
| P19 | Tissue | NA | 882.31 | 47.28% | 98.92% | 0.19% |
| P20 | Tissue | NA | 905.23 | 46.88% | 98.92% | 0.19% |
| P21 | Tissue | NA | 1072.84 | 51.06% | 98.91% | 0.31% |
| P22 | Tissue | NA | 597.74 | 46.36% | 99.19% | 0.77% |
| P23 | Tissue | NA | 775.4 | 37.31% | 98.74% | 0.27% |
| P24 | Tissue | NA | 670.78 | 45.54% | 98.15% | 0.42% |
| P25 | Tissue | NA | 815.91 | 38.87% | 98.25% | 0.90% |
| P26 | Tissue | NA | 753.26 | 38.91% | 99.29% | 0.20% |
| P27 | Tissue | NA | 673.79 | 38.59% | 97.88% | 0.89% |
| P28 | Tissue | NA | 616.39 | 38.93% | 99.26% | 0.20% |
| P29 | Tissue | NA | 957.53 | 51.08% | 98.60% | 0.81% |

| | | | | | | |
|---|---|---|---|---|---|---|
| P30 | Tissue | NA | 709.99 | 44.83% | 98.16% | 0.43% |
| P31 | Tissue | NA | 739.65 | 39.01% | 98.54% | 0.90% |
| P32 | Tissue | NA | 682.98 | 47.96% | 99.22% | 0.75% |
| P33 | Tissue | NA | 788.94 | 40.61% | 98.12% | 0.87% |
| P34 | Tissue | NA | 622.2 | 44.00% | 98.34% | 0.43% |
| P35 | Tissue | NA | 822.18 | 38.72% | 98.01% | 0.89% |
| P36 | Tissue | NA | 765.22 | 46.60% | 99.19% | 0.77% |
| P37 | Tissue | NA | 698.85 | 46.86% | 97.24% | 0.87% |
| P38 | Tissue | NA | 793.03 | 48.15% | 98.81% | 0.18% |
| P39 | Tissue | NA | 972.51 | 47.09% | 98.89% | 0.19% |
| P40 | Tissue | NA | 564.78 | 48.23% | 99.15% | 0.77% |
| P41 | Tissue | NA | 1061.51 | 53.45% | 99.67% | 0.30% |
| P42 | Tissue | NA | 583.63 | 46.78% | 97.42% | 0.81% |
| P43 | Tissue | NA | 548.37 | 49.00% | 99.01% | 0.78% |
| P44 | Tissue | NA | 655.04 | 41.73% | 98.83% | 0.79% |
| P45 | Tissue | NA | 565.4 | 46.76% | 97.43% | 0.81% |
| P46 | Tissue | NA | 742.83 | 46.21% | 98.21% | 0.42% |
| P47 | Tissue | NA | 636.9 | 49.58% | 99.22% | 0.75% |
| P48 | Tissue | NA | 916.06 | 45.96% | 98.84% | 0.76% |
| P49 | Tissue | NA | 707.35 | 49.82% | 97.58% | 0.87% |
| P50 | Tissue | NA | 648.29 | 42.03% | 98.85% | 0.79% |
| P51 | Tissue | NA | 1016.04 | 49.69% | 98.88% | 0.32% |
| P52 | Tissue | NA | 804.89 | 39.73% | 97.13% | 0.90% |
| P53 | Tissue | NA | 562.01 | 39.42% | 98.71% | 0.83% |
| P54 | Tissue | NA | 955.3 | 38.17% | 99.27% | 0.19% |
| P55 | Tissue | NA | 1471.42 | 48.76% | 99.68% | 0.80% |
| P56 | Tissue | NA | 1419.13 | 49.97% | 98.64% | 0.77% |
| P57 | Tissue | NA | 1045.3 | 51.11% | 98.77% | 0.31% |
| P58 | Tissue | NA | 961.05 | 50.11% | 99.57% | 0.30% |
| P59 | Tissue | NA | 452.48 | 45.65% | 99.08% | 0.40% |
| P60 | Tissue | NA | 647.85 | 46.36% | 98.14% | 0.42% |
| P61 | Tissue | NA | 587.73 | 44.19% | 99.18% | 0.39% |
| P62 | Tissue | NA | 664.24 | 41.68% | 98.96% | 0.80% |
| P63 | Tissue | NA | 649.93 | 46.32% | 97.14% | 0.87% |
| P64 | Tissue | NA | 635.05 | 39.91% | 98.53% | 0.83% |
| P65 | Tissue | NA | 525.36 | 39.36% | 98.17% | 0.85% |
| P66 | Tissue | NA | 574.41 | 42.65% | 98.29% | 0.82% |
| P67 | Tissue | NA | 684.79 | 39.66% | 98.72% | 0.82% |
| P68 | Tissue | NA | 668.27 | 44.54% | 99.08% | 0.40% |
| P69 | Tissue | NA | 709.19 | 51.00% | 99.25% | 0.74% |
| P70 | Tissue | NA | 610.98 | 44.46% | 99.05% | 0.39% |
| P71 | Tissue | NA | 625.57 | 47.37% | 97.46% | 0.80% |

*C01-03, healthy control; P01-71, patients with primary breast cancer.
